# Prognostic Value of ^18^F-FDG PET/CT Volume-Based Metabolic Parameters in Patients with Node-Negative Stage II Esophageal Squamous Cell Carcinoma

**DOI:** 10.3390/metabo12010007

**Published:** 2021-12-22

**Authors:** Daniel Hueng-Yuan Shen, Hung-Pin Chan, Fu-Ren Tsai, Chin Hu, Allan Yi-Nan Chen, Hung-Yen Chan, Che-Hsin Lee, Kuo-Pin Chuang, Ming-Hui Yang, Yu-Chang Tyan

**Affiliations:** 1 Department of Nuclear Medicine, Kaohsiung Veterans General Hospital, Kaohsiung 813, Taiwan; hyshen@vghks.gov.tw (D.H.-Y.S.); hpchan@vghks.gov.tw (H.-P.C.); frtsai@vghks.gov.tw (F.-R.T.); ghu@vghks.gov.tw (C.H.); J108217123@nkust.edu.tw (H.-Y.C.); 2 School of Medicine, University of California, Davis, Sacramento, CA 95817, USA; aychen@ucdavis.edu.tw; 3 Department of Biological Sciences, National Sun Yat-Sen University, Kaohsiung 804, Taiwan; chlee@mail.nsysu.edu.tw; 4 Graduate Institute of Animal Vaccine Technology, College of Veterinary Medicine, National Pingtung University of Science and Technology, Pingtung 912, Taiwan; kpchuang@g4e.npust.edu.tw; 5 Department of Medical Education and Research, Kaohsiung Veterans General Hospital, Kaohsiung 813, Taiwan; 6 Center of General Education, Shu-Zen Junior College of Medicine and Management, Kaohsiung 821, Taiwan; 7 Department of Medical Imaging and Radiological Sciences, Kaohsiung Medical University, Kaohsiung 807, Taiwan; 8 Department of Nuclear Medicine, Kaohsiung Medical University Hospital, Kaohsiung 807, Taiwan; 9 Graduate Institute of Medicine, College of Medicine, Kaohsiung Medical University, Kaohsiung 807, Taiwan; 10 Institute of Medical Science and Technology, National Sun Yat-Sen University, Kaohsiung 804, Taiwan; 11 Department of Medical Research, Kaohsiung Medical University Hospital, Kaohsiung 807, Taiwan; 12 Center for Cancer Research, Kaohsiung Medical University, Kaohsiung 807, Taiwan; 13 Research Center for Environmental Medicine, Kaohsiung Medical University, Kaohsiung 807, Taiwan

**Keywords:** esophageal squamous cell carcinoma, metabolic tumor burden, overall survival, prognostic value, PET/CT

## Abstract

Esophageal squamous cell carcinoma (ESCC) is a major cancer prevalent in Asian males. Pretreatment tumor burden can be prognostic for ESCC. We studied the prognostic value of metabolic parameters of 2-deoxy-2-[^18^F] fluoro-D-glucose positron emission tomography/computed tomography (^18^F-FDG PET/CT) and the serum squamous cell carcinoma antigen (SCC-Ag) level in node-negative stage II ESCC patients. Eighteen males underwent staging evaluation were included. The volume-based metabolic parameters derived from ^18^F-FDG PET/CT, including metabolic tumor volume (MTV) and total lesion glycolysis (TLG), were obtained using the PET Volume Computer Assisted Reading application. The Spearman correlation coefficients were calculated to assess the relationship between metabolic parameters and pretreatment serum SCC-Ag levels. Based on the 5-year follow-up, patients were sub-divided into the demised and the stable groups. Potential prognostic value was assessed by independent *t*-test and the Mann–Whitney U test. The association of overall survival was assessed using univariate and multivariate Cox regression analyses. The demised group showed significant higher values in serum SCC-Ag, as well as in MTV and TLG, but not SUVmax and SUVmean. The SUVmax, MTV, TLG, and serum SCC-Ag showed significant association with overall survival. Our findings suggest potential usage of pretreatment volume-based metabolic parameters of ^18^F-FDG PET/CT and serum SCC-Ag as prognostic factors for node-negative stage II ESCC patients.

## 1. Introduction

Esophageal cancer is the eighth common cancer worldwide [1] and the fifth prevalent cancer in Taiwan [2]. ESCC and esophageal adenocarcinoma (EAC) are the two major histopathological subtypes of esophageal cancer. While EAC is more common in North America and certain parts of Europe [3], ESCC constitutes about 90% of esophageal cancers in Eastern Asia [4]. The treatment options for stage II ESCC patients include esophagectomy alone, chemoradiotherapy (CRT) alone, or combination of esophagectomy and CRT [5].

Integrating clinical information of primary tumor, as well as nodal and distant metastasis, the TNM staging system is the overall most reliable prognostic factor for cancers. Node metastasis has been shown to be a major prognostic factor for esophageal cancer [6]. However, largely based on tumor size and anatomy, the TNM staging system lacks tumor metabolic information and, unavoidably, contains major prognostic discrepancy when comparing patients with small-sized, metabolically aggressive tumors to patients with large-sized, metabolically indolent tumors. Despite recent medical advances, according to the nationwide population-based study of Taiwan, the five-year overall survival for stage II esophageal cancer was only about 28% [2]. Information in reliable prognostic and predictive factors is urgently needed in early, non-metastatic esophageal cancer.

The 2-deoxy-2-[^18^F] fluoro-D-glucose positron emission tomography/computed tomography (^18^F-FDG PET/CT) can provide both anatomic and metabolic information and has become an excellent staging tool for whole-body evaluation of cancer patients [7]. ^18^F-FDG PET/CT is also a valuable tool to assess responses after therapeutic interventions with chemotherapy and/or radiotherapy [8,9]. Among the metabolic parameters derived from ^18^F-FDG PET/CT, the maximum standardized uptake value (SUVmax) and mean standardized uptake values (SUVmean) are most frequently used in routine clinical settings to monitor changes in tumor metabolic activity before and after treatment. Recently, clinical applications of volume-based metabolic parameters derived from ^18^F-FDG PET/CT have been explored in various clinical settings [10,11,12,13,14]. Among the volume-based metabolic parameters, the metabolic tumor volume (MTV) delineates the volume of tumor with increased glycolytic activity, and the total lesion glycolysis (TLG) is calculated by multiplying MTV by SUVmean of the delineated tumor. Recent studies have shown potential usage of MTV and TLG in predicting clinical outcomes for different cancers [11,12,13,14].

Serum squamous cell carcinoma antigen (SCC-Ag) is one of tumor-associated antigens related to squamous cell carcinoma. Molecular biology studies demonstrate that SCCA belongs to the super family of serine protease inhibitors and functions as suicide substrates for cellular proteases. Serum SCC-Ag has been adapted as a marker in squamous cell carcinoma of the head and neck, lung, and esophagus [15]. The current study aimed to evaluate the prognostic value of ^18^F- FDG PET/CT-derived volume-based metabolic parameters and serum SCC-Ag in patients with ESCC staging II.

## 2. Results

### 2.1. Patient Characteristics

Table 1 showed characteristics of the 18 node-negative stage II ESSC patients in our study, including seven patients with T2N0 (38.9%) and 11 patients with T3N0 (61.1%) were identified. The median age of these patients was 62 years old (range 49 to 83), including four patients below 50 years of age, four patients between 50 and 60 years of age, seven patients between 60 and 70 years of age, and two patients older than 70 years of age. Interestingly, all 18 patients were of male gender, with 14 patients with history of cigarette smoking (77.8%) and 12 patients with history of heavy alcohol consumption (69.4%). Among the 18 patients, 11 patients underwent CRT (61.1%) alone, five patients underwent esopahgectomy (27.7%) alone, and two patients (11.1%) received both CRT and esopahgectomy.

### 2.2. Comparison of Serum SCC-Ag Level and ^18^F-FDG PET/CT-Derived Metabolic Parameters in the Demised versus Stable Prognostic Groups Patients with Stage II ESCC

The Spearman’s rank correlation coefficients were calculated to assess the relationship between different tumor metabolic parameters derived from ^18^F-FDG PET/CT and serum SCC-Ag in our patient. As shown in Table 2, SUVmax showed strong positive correlation with SUVmean (Spearman’s ρ = 0.962; 95% confidence interval [CI], 0.96–0.95), moderate positive correlation with MTV (Spearman’s ρ = 0.483; 95% confidence interval [CI], 0.42–0.59), near strong positive correlation with TLG (Spearman’s ρ = 0.682; 95% confidence interval [CI], 0.58–0.79) and near strong positive correlation with serum SCC-Ag (Spearman’s ρ = 0.646; 95% confidence interval [CI], 0.52–0.73). SUVmean showed moderate positive correlation with MTV (Spearman’s ρ = 0.527; 95% confidence interval [CI], 0.48–0.57), near strong positive correlation with TLG (Spearman’s ρ = 0.733; 95% confidence interval [CI], 0.67–0.84) and near strong positive correlation with serum SCC-Ag (Spearman’s ρ = 0.607; 95% confidence interval [CI], 0.50–0.69). MTV showed strong positive correlation with TLG (Spearman’s ρ = 0.922; 95% confidence interval [CI], 0.89–0.96) and near strong positive correlation with serum SCC-Ag (Spearman’s ρ = 0.737; 95% confidence interval [CI], 0.67–0.82).

Based on patient prognosis with 5-years follow-up, the 18 node-negative stage II ESSC patients were sub-divided into the demised (*n* = 8) and the stable (*n* = 10) groups. The independent *t*-test was performed to evaluate serum SCC-Ag level and ^18^F-FDG PET/CT-derived metabolic parameters in the two prognostic groups of patients. As shown in Table 3, the values of SUVmax (20.36 ± 12.64 vs. 11.59 ± 5.71) and SUVmean (10.7 ± 6.62 vs. 6.82 ± 3.05) showed no statistical difference between the demised group and the stable group. In contrast, significantly higher value of serum SCC-Ag level (4.29 ± 4.24 vs. 1.17 ± 0.52; *p* < 0.05), MTV (24.42 ± 18.26 vs. 7.59 ± 8.39; *p* < 0.05), and TLG (10.7 ± 5.62 vs. 6.82 ± 3.05; *p* < 0.05) were observed in the demised group comparing to the stable group (Table 3). Given the relative small sample size, the Mann–Whitney U test was performed to evaluate these parameters (Table 4). Again, statistical significant differences were detected for MTV and TLG (*p* < 0.01), but not for SUVmax or SUVmean, between the two prognostic patient groups (Table 4). However, the difference in serum SCC-Ag level became statistically insignificant (*p* = 0.055) with the Mann-Whitney U test analysis (Table 4).

**Table 3:** Comparison of the tumor metabolic parameters of demised versus stable patients by independent *t*-test.

Parameter	Demised (*n* = 8)	Stable (*n* = 10)	*p* Value
SUVmax	20.36 ± 12.64	11.59 ± 5.71	0.101
SUVmean	10.70 ± 5.62	6.82 ± 3.05	0.109
MTV	24.42 ± 18.26	7.59 ± 8.39	0.038 *
TLG	311.53 ± 362.47	48.79 ± 47.96	0.036 *
SCC-Ag	4.29 ± 4.24	1.17 ± 0.52	0.034 *

**Figure 1:**
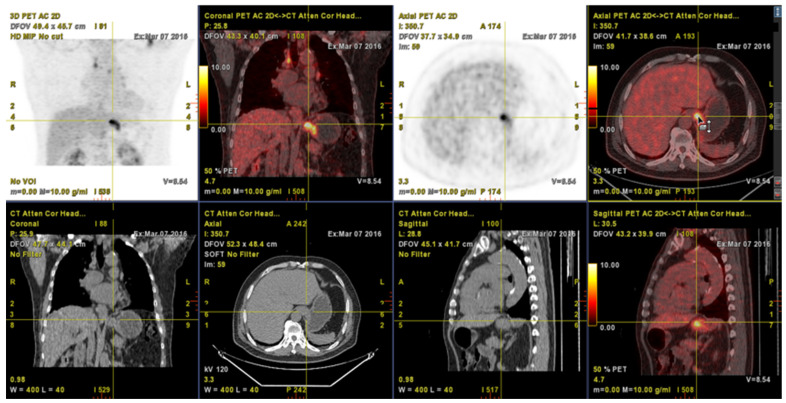
A 78 y/o male patient was diagnosed cT2N0M0, stage II on pre-treatment ^18^F-FDG PET/CT. The small tumor mass with FDG hot uptake was noted on ^18^F-FDG PET/CT (yellow color cross indicator). Metabolic parameters showed SUVmax: 7.61, SUVmean: 4.65, MTV: 11.07 cm^3^, and TLG: 51.41g/mL cm^3^ by the PET VCAR. He underwent CRT treatment and expired 3.9 months later due to disease progression.

* *p* < 0.05; Figure 1 showed a pretreatment whole-body ^18^F-FDG PET/CT of an example case of 78 years old male patient with stage IIA (cT2N0M0) ESCC. With no smoking history, he was found to have a small-sized distal esophageal tumor with relatively high FDG uptake (Figure 1). As shown, the values of his ^18^F-FDG PET/CT-derived metabolic parameters are: SUVmax: 7.61, SUVmean: 4.65, MTV: 11.07 cm^3^ and TLG: 51.41 g/mL cm^3^ by the PET VCAR. He underwent CRT and, unfortunately, deceased in 3.9 months due to rapid disease progression.

### 2.3. Comparison of Serum SCC-Ag Level and ^18^F-FDG PET/CT-Derived Metabolic Parameters in Surgery versus CRT Patient Groups Patients with Stage II ESCC ESCC

Serum SCC-Ag level and ^18^F-FDG PET/CT-derived metabolic parameters in surgery alone (*n* = 5) versus CRT alone (*n* = 11) patients were evaluated. Comparing the surgery group to the CRT group, the independent *t*-test showed no significant difference in SUVmax (13.5 ± 6.2 vs. 16.4 ± 11.1), SUVmean (7.24 ± 3.66 vs. 8.91 ± 4.54), MTV (11.35 ± 7.91 vs. 13.45 ± 10.9), TLG (59.81 ± 22.73 vs. 143.55 ± 169.73), or serum SCC-Ag level (2.24 ± 2.46 vs. 2.01 ± 2.48) (data not shown).

### 2.4. Relationships between Serum SCC-Ag Level and ^18^F-FDG PET/CT-Derived Metabolic Parameters with Overall Survival (OS) in ESCC Staging II Patients

**Figure 2:**
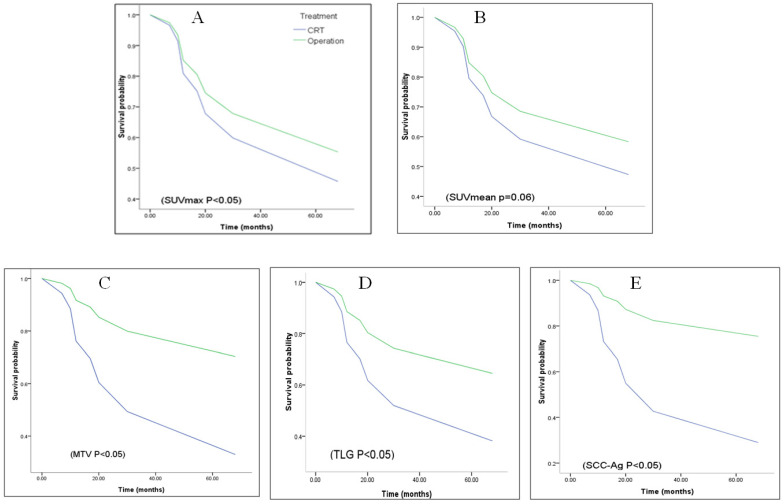
Kaplan–Meier curves of different tumor metabolic parameters for overall survival of patients. (**A**) SUVmax, (**B**) SUVmean, (**C**) MTV, (**D**) TLG, and (**E**) SCC-Ag.

The association of various metabolic parameters and serum SCC-Ag with patient overall survival was evaluated by univariate and multivariate Cox regression analyses. As shown in Table 5, SUVmax, MTV, TLG, and serum SCC-Ag, but not SUVmean, showed a significant association with OS in our studied node-negative stage II ESCC patient. Additional univariate or multivariate analyses did not show any significant association of age, smoking and treatment with OS (data not shown). Kaplan–Meier survival analysis revealed survival probability of CRT (solid line) less than operation with several parameters that showed in Figure 2. 

## 3. Discussion

The current TNM staging system for esophageal cancer is based on only anatomic finding of tumor, without tumor metabolic information. The ^18^F-FDG PET/CT is a powerful noninvasive modality that can provide not only anatomic, but also metabolic information of tumor. SUVmax is the most common ^18^F-FDG PET/CT metabolic parameter routinely used in the clinic. Recently, there are increasing evidence to support usage of different ^18^F-FDG PET/CT-derived metabolic parameters in assessing pretreatment extent of disease for various cancers [14,16,17]. Different from SUVmax measuring on a single voxel and may not reflect the whole tumor metabolism, volume-based metabolic parameters such as MTV and TLG are volumetric measurements incorporated with metabolic activity throughout the entire tumor. Lately, MTV and TLG have been shown of prognostic value to predict clinical outcomes of cancer patients [11,12,18,19]. Serum SCC-Ag is a tumor marker and elevated serum SCC levels are known to be associated with advanced tumor stage, poor treatment response, and increased risk of tumor recurrence [20]. As shown in the current study, positive correlations were found among various ^18^F-FDG PET/CT-derived metabolic parameters and serum SCC-Ag for node-negative stage II ESCC patients. Among them, the SUVmax and SUVmean showed moderate positive correlation with MTV, and near strong positive correlation with TLG and serum SCC-Ag.

The current study investigated prognostic value of ^18^F-FDG PET/CT-derived metabolic parameters and serum SCC-Ag in early-stage ESCC patients. A total of 18 node-negative stage II ESCC patients with sufficient follow-up of 5-years was sub-divided into the demised (poor prognosis) and the stable (good prognosis) group. Our study showed patients in the demised group have statistically significant higher values than the stable group in MTV and TLG, but not in SUVmax and SUVmean. Previously, Mantziari et al. reported higher values of baseline metabolic parameters of ^18^F-FDG PET/CT were significantly related to the tumor location and presence of advanced T3 and T4 stages in esophageal cancer [14]. In their study, SUVmax > 8.25 g/mL, TLG > 41.7, and MTV > 10.7 cm^3^ were noted in their cT/4 stage cases. They also defined SUVmax ≥ 12.7 g/mL that can be predicted early recurrence and poor disease-free survival. In a study by Wang et al., pretreatment SUVmean was shown to be a better independent predictor of treatment response than SUVmax, MTV, and TLG in patients with locally advanced ESCC treated with concurrent chemoradiotherapy [21]. The subtle discrepancies between our findings and others can be due to differences in studied patient populations and variations in treatment modalities. Solid nodule type, poor histological grade, and larger nodule size have been reported to be associated with higher values of SUV, MTV, and TLG in stage I lung adenocarcinoma [22]. Taken together, the higher MTV and TLG values in the demised group patients of our study could be related to the poor pathological grades or other features of their tumors. Further studies are needed to establish correlations between pathological grades and ^18^F-FDG PET/CT-derived volume-based metabolic parameters.

Our study showed serum SCC-Ag level significant increased (4.29 ± 4.24 vs. 1.17 ± 0.52; *p* < 0.05) in the demised group patients, as compared to the stable group patients. It also correlated to ^18^F-FDG PET/CT-derived parameters of the main tumor in ESCC stage II patients. In Cox models, it was significantly associated with OS. However, the level of SCC-Ag sometimes is also observed at an elevated level in lung, cervix, head and neck cancer or benign disease, including skin disease, pelvic inflammatory disease, cystitis and renal failure [23,24,25]. Therefore, it is a limitation for serum SCC-Ag level screening in clinical routine, though it is a convenient test for pre-treatment or post-treatment surveillance of patients.

Another aim of this study was to investigate the association of various ^18^F-FDG PET/CT-derived tumor metabolic parameters and serum SCC-Ag with the overall survival in node-negative stage II ESCC patients. Based on the univariate and multivariate Cox regression analyses, serum SCC-Ag, SUVmax, MTV, and TLG were identified to be independent predictors of OS. SUVmean was the only exception that did not shown statistical significant association with OS by univariate (*p* = 0.057) and multivariate analyses (*p* = 0.060). In this regard, Tamandl et al. reported no correlation between various metabolic parameters and OS in 38 patients with unresectable or metastatic ESCC who had ^18^F-FDG PET/CT prior to palliative treatment [26]. Albeit, in the same study, MTV was shown to be predictive for OS in 33 patients with unresectable or metastatic esophageal adenocarcinoma (EAC) [26]. The discrepancy of MTV being predictive factor of OS between node-negative early-stage ESCC and advanced-stage ESCC patients is intriguing and demands additional investigations. Nevertheless, our study also identified SCC-Ag, SUVmax, and TLG being independent predictors of OS in early-stage ESCC patients. Another issue of partial volume effect (PVE) for SUVmax measurements was reported by Hatt and et al. [27]. They mentioned PVE correction did not add any value in volume-based metabolic parameters of ^18^F-FDG PET for prognosis of esophageal cancer, although PVE correction may increase the levels of SUVmax and SUVmean.

The difference of our study with other studies mainly included (1) to evaluate and compare the SCC-Ag level and ^18^F-FDG PET/CT-derived tumor metabolic parameters of pre-treatment tumor burden of demised and stable patients in early-stage ESCC. There were no factors of nodal metastasis, distant metastasis or tumor recurrence in our study; (2) to evaluate the prognostic value of SCC-Ag level and ^18^F-FDG PET/CT-derived tumor metabolic parameters in early-stage ESCC patients. To our knowledge, this is the first study discussing the relationship and prognostic value between serum SCC-Ag with ^18^F-FDG PET/CT-derived tumor metabolic parameters of tumor burden in early-stage ESCC patients. We also concluded the parameters and prognostic value of tumor burden in different clinical status. A systematic review and meta-analysis article was reported by Han and et al. [28]. In this article, they employed different staging and patient-specific treatment from several published articles for discussion. They presented that higher MTV and TLG values on pre-treatment ^18^F-FDG PET/CT of esophageal patients were at higher risk of adverse events or death. This finding is similar to ours. Another similar result of volume-based metabolic parameters was found as having significant prognostic factors in OS with esophageal cancer and our ESCC stage II patients. In our study, we also found serum SCC-Ag and SUVmax of pre-treatment ^18^F-FDG PET/CT that could be prognostic biomarkers in ESCC stage II patients. However, SUVmean was not a significant prognostic biomarker in our study.

There are limitations for the current study. Firstly, this is a retrospective study of only node-negative stage II ESCC patients that is limited by small sample size and patient selection bias. For example, all our patients are of male gender. Small sample size may cause poor statistical power and increase the error of study. Patient selection bias may affect the external validity of the study. The results are from a selected group and may not be generalized to all patients, not even to all males. Secondly, patients with tumor recurrence or nodal metastasis were excluded from the study. Therefore, our results cannot be applied for these patient groups. Thirdly, our patients received different treatment modalities, different regimens of chemotherapy and likely varying doses of radiotherapy, which could have affected the outcome.

## 4. Materials and Methods

### 4.1. Patients

From 1 January 2015 to 31 December 2016, there were 117 patients with biopsy proved ESCC underwent pre-treatment ^18^F-FDG PET/CT examination in our institution. After chart review, 18 patients with node-negative stage II ESSC, including 7 patients with T2N0 (38.9%) and 11 patients with T3N0 (61.1%) were identified. Patients with history of previous cancer, prior esophageal surgery, evidence of lymph node metastasis by ^18^F-FDG PET/CT or other examination and histological type other than SCC were excluded from the study. Based on clinical information with 5 years of follow-up, patients were sub-divided into the demised and the stable prognostic groups. The treatment of patients was decided by a multidisciplinary team, consisting with surgeons, medical oncologists and radiation oncologists. Serum samples were obtained from peripheral blood of patients, and then centrifuged for 10 min at 1300× *g*. The serum SCC-Ag levels were measured and recorded by Chemiluminescent Microparticle Immunoassy (CMIA) (ABBOTT GMBH & CO.KG, Wiesbaden, Germany). Data analysis was conducted from the patent product of Abbott Architect SCC (Tokyo, Japan). The study design was approved by the Institutional Review Board in our hospital (KSVGH 21-CT14-07). The characteristics of the enrolled patients are given in Table 1.

### 4.2. ^18^F-FDG PET/CT Imaging

All patients were suggested to fast for at least 6 h before ^18^F-FDG PET/CT imaging. An intravenous catheter was placed before the radiopharmaceutical agent injection, and patients’ blood glucose levels were measured before the tracer injection for ensuring good PET/CT imaging quality (adequate blood glucose level <150 mg/dL). Each patient was administrated with 370–555 MBq of ^18^F-FDG according to the body weight (7.03 MBq/kg). After injection of the ^18^F-FDG tracer, patients then underwent whole-body ^18^F-FDG PET/CT (Discovery ST-16; GE Healthcare, Milwaukee, WI, USA) from the head to the upper thigh in a supine position. A delayed image might be obtained while necessary. CT scanning was performed prior to acquisition of the PET imaging by using the subsequent parameters: 0.6 s per rotation, 120 kV, 100 mA, and 3.75 mm thick slices. An ordered subset expectation maximization iterative reconstructed algorithm was used for attenuation-corrected PET images reconstruction. The fusion imaging of PET and CT were obtained on a Xeleris image display and processing platform (GE Healthcare, Milwaukee, WI, USA).

### 4.3. Imaging Analysis

The PET, CT, and fused PET/CT images of each patient were independently reviewed and interpreted by three experienced nuclear medicine physicians. The FDG-avid esophageal tumor detected by PET was fused to the corresponding lesion and anatomical location on CT scan. Standardized uptake values (SUVs) were used as the metric for metabolic tumor quantification with ^18^F-FDG PET. The SUV was measured semi-automatically by SUV tools obtained in the Xeleris software (Version 4.0) as follows: SUV = activity in the region of interest (Bq/g)/(injected dose(Bq)/body weight (g)). The SUVmax is the highest SUV detected for the tumor. The metabolic tumor volume (MTV; cm^3^) is defined as total tumor volume with an SUV of ≥2.5. Total lesion glycolysis (TLG; g/mL cm^3^) is calculated by multiplying MTV by SUVmean of the delineated tumor. The PET VCAR (volume computer assisted reading; GE Healthcare) was used for imaging analysis. After drawing a cuboid volume of interest (VOI) covering the tumor, the software then automatically drew the FDG uptake of tumor margin according to the specific SUV threshold. MTV and TLG then were automatically computed and measured by the PET VCAR application (Advanced workstation 4.4, GE Medical System, Milwaukee, WI, USA).

### 4.4. Statistical Analysis

The results are shown as mean ± standard deviation. The Spearman correlation coefficients were calculated to assess the relationship between different tumor metabolic parameters derived from ^18^F-FDG PET/CT and serum SCC-Ag in our patients. Statistical significance of different ^18^F-FDG PET/CT metabolic parameters and serum SCC-Ag level between the demised and stable prognostic groups of patients was assessed by using independent *t*-test and Mann–Whitney U test. The univariable or multivariable Cox regression model with Breslow approximation was used to determine the hazards ratio of parameters. A *p*-Value of <0.05 was considered statistically significant. All statistical analysis was performed using the SPSS software package (version 17.0, Chicago, IL, USA).

## 5. Conclusions

In comparison to the stable prognostic group, patients in the demised prognostic group showed significant higher values in serum SCC antigen (SCC-Ag) level, as well as in the volume-based metabolic parameters MTV and TLG (*p* < 0.05), but not SUVmax (*p* = 0.10) and SUVmean (*p* = 0.11). By univariate and multivariate Cox regression analyses, values of SUVmax, MTV, TLG, and serum SCC-Ag, but not SUVmean, showed significant association with overall survival of studied patients. Our findings suggest potential usage of pretreatment volume-based metabolic parameters of ^18^F-FDG PET/CT and serum SCC-Ag as prognostic factors for node-negative stage II ESCC patients.

## Figures and Tables

**Table 1:** Characteristics of the enrolled patients in this study.

Patient Characteristics	Number (%) or Mean (SD)
*Demographic characteristics*	
Age (year)	62 (49–83)
Male	18 (100%)
Smoking	14 (77.8%)
Alcohol consumption	12 (69.4%)
*Clinical characteristics*	
T stage	
T2	7 (38.9%)
T3	11 (61.1%)
Treatment	
CRT only	11 (61.1%)
Operation only	6 (33.3%)
CRT + operation	2 (11.1%)

**Table 2:** Spearman’s rank correlation coefficients between different tumor metabolic parameters and SCC-Ag.

Parameter	ρ/*p* Value	SUVmax	SUVmean	MTV	TLG
SUVmax	ρ	/	0.962	0.483	0.682
	*p* Value	/	0.000	0.042	0.002
SUVmean			/	0.527	0.733
			/	0.025	0.001
MTV				/	0.922
				/	0
TLG					/
					/
SCC-Ag		0.646	0.607	0.737	0.843
		0.004	0.008	0.000	0.000

SUV_max_ = maximum standardized uptake value, SUV_mean_ = mean standardized uptake value, MTV = metabolic tumor volume, TLG = total lesion glycolysis, SCC-Ag = squamous cell carcinoma antigen.

**Table 4:** Comparison of the tumor metabolic parameters of demised versus stable patients by Mann–Whitney U test analysis.

Parameter	Mean rank (Demised, *n* = 8)	Mean rank (Stable, *n* = 10)	*Z* Value	*p* Value
SUVmax	12	7.5	−1.78	0.083
SUVmean	11.63	7.8	−1.51	0.146
MTV	13.13	6.6	−2.57	0.009 **
TLG	13.38	6.4	−2.75	0.006 **
SCC-Ag	12.25	7.3	−1.96	0.055

** *p* < 0.01.

**Table 5:** Univariate and multivariate Cox regression analysis for overall survival.

Variable	Hazard Ratio (95% CI)	*p* Value
***Univariate analysis***		
**SUVmax**	1.112 (1.019–1.213)	0.017
**SUVmean**	1.173 (0.995–1.383)	0.057
**MTV**	1.035 (1.004–1.067)	0.029
**TLG**	1.002 (1.000–1.003)	0.043
**SCC-Ag**	1.127 (1.030–1.437)	0.021
***Multivariate analysis***		
**SUVmax**	0.126 (1.023–1.248)	0.016
**SUVmean**	1.211 (0.992–1.478)	0.06
**MTV**	1.053 (1.007–1.101)	0.024
**TLG**	1.002 (1.000–1.005)	0.043
**SCC-Ag**	1.368 (1.040–1.2799)	0.025

## Data Availability

The data presented in this study is available on request from the corresponding author. The data is not publicly available due to its proprietary nature.
